# Exometabolomics Reveals Metabolic Modulation Induced by Chia Oil Supplementation in Adipose Tissue Explants From Obese Mice

**DOI:** 10.1002/lipd.70066

**Published:** 2026-05-31

**Authors:** Gilson Costa dos Santos, Agatha Assis‐Ferreira, Gabrielly Muniz‐Cassuce, Tatiana El Bacha, Christina Barja‐Fidalgo, Simone Vargas da Silva

**Affiliations:** ^1^ Laboratório de Metabolômica, Departamento de Genética Instituto de Biologia Roberto Alcântara Gomes, Universidade do Estado do Rio de Janeiro Rio de Janeiro Brazil; ^2^ Laboratório de Farmacologia Celular e Molecular, Departamento de Biologia Celular Instituto de Biologia Roberto Alcântara Gomes, Universidade do Estado do Rio de Janeiro Rio de Janeiro Brazil; ^3^ Núcleo de Estudos Com Bioativos, Mitocôndria e Metabolismo da Placenta, Instituto de Nutrição Josué de Castro Universidade Federal do Rio de Janeiro Rio de Janeiro Brazil

**Keywords:** adipose tissue, chia oil, exometabolomics, glucose metabolism, NMR‐based metabolomics, obesity

## Abstract

Obesity impairs adipose tissue metabolism and dietary interventions capable of modulating these alterations remain insufficiently explored. To investigate depot‐specific metabolic adaptations in subcutaneous (SAT) and epididymal adipose tissue (EAT) in response to chia oil supplementation. Male C57BL/6J mice received a control diet (C group) or a high‐fat diet (H group) for 8 weeks, followed by 5 weeks of chia oil supplementation (1.5% w/w; 1.5 g/100 g diet; CC and HC groups). Exometabolomic profiling of SAT and EAT explants was performed using ^1^H‐NMR, accompanied by targeted gene‐expression analyses. Chia oil supplementation reduced adipocyte hypertrophy and improved glucose tolerance in obese mice without altering body or adipose tissue mass. Obesity markedly decreased l‐histidine secretion in SAT and this effect was not modified by chia oil supplementation. In obese SAT, chia oil reduced l‐valine and l‐proline secretion and increased l‐threonine levels, while it increased lactate secretion in obese EAT. Obesity did not significantly affect acetate secretion, although chia oil supplementation resulted in a depot‐specific variation exclusively in the HC group. Correlation analysis revealed that chia oil modified metabolic interaction networks in both adipose depots. Chia oil supplementation induced depot‐specific metabolic adjustments in SAT and EAT, highlighting the plasticity of adipose tissue and the potential of dietary lipids to modulate adipose metabolic networks.

## Graphical abstract

The experimental design and the exometabolomic findings obtained by ^1^H‐NMR analysis of conditioned media from subcutaneous (SAT) and epididymal adipose tissue (EAT) explants of control and obese mice supplemented with chia oil (1.5% (w/w)). Following 8 weeks of control or high‐fat diet and 5 weeks of chia oil supplementation, SAT and EAT explants were collected and their metabolites profiled by NMR. Chia oil induced depot‐specific metabolic adaptations, including reduced secretion of histidine, valine, and proline and increased threonine release in SAT, as well as elevated lactate secretion in EAT. Correlation analysis further revealed chia‐dependent reorganization of metabolic interaction networks, highlighting coordinated shifts in amino acid and glycolytic pathways. The graphical abstract depicts this workflow and the principal metabolic changes observed.

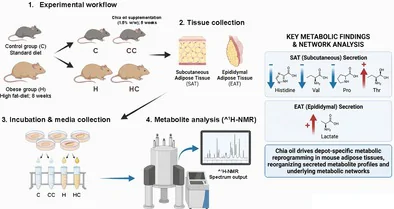

**Abbreviations**
ALA: α‐linolenic acid
AUC: area under the curve
BCAA: branched‐chain amino acid
C: control diet
CC: control diet supplemented with chia oil
CM: conditioned medium
CT: cycle threshold
DMEM: Dulbecco's modified eagle medium
EAT: epididymal adipose tissue
ECM: extracellular matrix
FDR: false discovery rate
FFA: free fatty acid
GLUT4: glucose transporter type 4
H: high‐fat diet group
HC: high‐fat diet supplemented with chia oil
HFD: high‐fat diet
ipGTT: intraperitoneal glucose tolerance test
IRS‐1: insulin receptor substrate‐1
LDH: lactate dehydrogenase
MAFLD: metabolic‐dysfunction‐associated fatty liver disease
MCT: monocarboxylate transporter
MS: manuscript
NMR: nuclear magnetic resonance
PBS: phosphate‐buffered saline
PCA: principal component analysis
PVAT: perivascular adipose tissue
qPCR: quantitative polymerase chain reaction
RNA: ribonucleic acid
SAT: subcutaneous adipose tissue
SEM: standard error of the mean
TCA cycle: tricarboxylic acid cycle
VAT: visceral adipose tissue

## 1 Introduction

The high worldwide prevalence of obesity has emerged as a global public health problem, contributing to type 2 diabetes mellitus, metabolic‐dysfunction‐associated fatty liver disease (MAFLD) and cardiovascular diseases such as hypertension, heart disease, and stroke (WHO 2025; Ahmed and Mohammed 2025). Research on metabolic changes in adipose tissue—specifically visceral (VAT) and subcutaneous adipose tissue (SAT)—is increasing, but the mechanisms underlying adipose tissue dysfunction in obesity and associated diseases are not fully understood (Brand et al. 2021; Neeland et al. 2013). Understanding these alterations is crucial for developing effective prevention and treatment strategies.

One potential intervention is dietary supplementation with chia oil ( *Salvia hispanica* L), which is rich in the ω‐3 fatty acid α‐linolenic acid (ALA). Our group previously demonstrated that chia oil supplementation improves insulin sensitivity in obese mice, promoting IRS‐1, AKT activation, and GLUT4 translocation in skeletal muscle (Fonte‐Faria et al. 2019). We also showed that chia oil supplementation induces a shift in body fat and lean mass in obese animals (de Souza et al. 2020). Moreover, chia oil supplementation ameliorated symptoms of metabolic syndrome in obese mice (de Souza et al. 2020).

Recent studies profiling the metabolic characteristics of adipose tissue in obese mice revealed alterations in lipid metabolism, inflammation markers, and adipokine secretion that contribute to the development and progression of obesity‐related diseases (Sun et al. 2011; Wang et al. 2023).

Adipose tissue plays a crucial role in metabolite exchange, particularly glucose and free fatty acids (FFAs). Glucose uptake, driven by insulin after meals, supports ATP production and fatty acid synthesis. Adipocytes take up FFAs from circulating lipoproteins via lipoprotein lipase, which are then either oxidized or stored as triglycerides (Morigny et al. 2021). Adipose tissue also consumes amino acids for protein synthesis and acetate for lipid production. During fasting, adipose tissue releases FFAs and glycerol from triglyceride breakdown to supply energy to other tissues, while lactate and branched‐chain amino acids (BCAAs) are released and can influence systemic metabolism and inflammation (Broskey et al. 2024; Feng et al. 2022; Burrill et al. 2015; Herman et al. 2010). The metabolic activity of adipose tissue is affected by nutritional status, depot location, and overall health, highlighting its dynamic role in energy balance and metabolic homeostasis (Sakers et al. 2022).

In this study, we examined for the first time the effects of chia oil supplementation on metabolite uptake and secretion using ^1^H‐NMR‐based exometabolomics in visceral and subcutaneous adipose tissue explants from obese mice. Our findings contribute to understanding depot‐specific metabolic adaptations to dietary lipid quality in obesity and identify pathways potentially underlying chia oil‐driven modulation of adipose exometabolism. This work may help inform dietary strategies for individuals at risk of obesity‐associated metabolic dysfunction.

## 2 Methods

### 2.1 Animals, Diet and Chia Oil Supplementation

All animal procedures were approved by the Institutional Committee for Ethics in Animal Use (CEUA) of the State University of Rio de Janeiro (UERJ; protocol number 016/2021) and followed NIH guidelines for animal care. Male C57BL/6J mice (3–4 weeks old) were obtained from the Central Animal House Services and housed at a controlled temperature under a 12 h light/12 h dark cycle. Animals were maintained in ventilated mini‐isolator cages (Alesco, São Paulo, Brazil) (*n* = 5 mice/cage) and were fed either a control diet (C: 396 kcal/100 g, 13% energy from fat) or a high‐fat diet (H: 470 kcal/100 g, 45% energy from fat) for 8 weeks. Diet composition (ingredients and macronutrient distribution) is detailed in Table S1. After 8 weeks, each dietary group was subdivided to continue the same base diet with or without chia oil for an additional 5 weeks, generating four experimental groups: C, CC, H, and HC. Chia oil was incorporated into the diet at 1.5 g/100 g of diet (1.5% (w/w)). Diet composition and fatty‐acid profiles are provided in Tables S1 and S2. To minimize lipid oxidation, diets containing chia oil were prepared and replaced in the cages every 3 days and were stored protected from light at 4°C until use. The ω‐6:ω‐3 ratios were 9.4 (C), 2.95 (CC), 11.52 (H) and 4.1 (HC), indicating that chia oil supplementation lowered the ω‐6:ω‐3 ratio in both CC and HC diets. The chia oil (Equaliv Pharma Ind, SP, Brazil) was cold‐pressed and consisted of approximately 60% α‐linolenic acid, 20% linoleic acid and 10% oleic acid. Chia oil was stored in an amber container, protected from light, at 4°C and used within 2 weeks after opening to minimize oxidation. Body weight and food intake were recorded throughout the experiment.

### 2.2 Blood Glucose Levels

Blood glucose was measured in conscious mice after 6 h fast using an AccuCheck Active glucometer (Roche Diagnostics, Mannheim, Germany) and compatible test strips. The tail was gently warmed and disinfected, a small lateral tail‐vein nick was made with a sterile lancet, the first drop was discarded, and the next drop was applied to the test strip according to the manufacturer's instructions. Values (mg/dL) were recorded immediately, and bleeding was stopped by gentle compression.

### 2.3 Intraperitoneal Glucose Tolerance Test (ipGTT)

After 8 h fasting, basal glycemia was measured. Mice received an ip injection of glucose (2.0 g/kg) and glycemia was monitored at 30 min intervals for up to 120 min using an AccuCheck Active glucometer.

### 2.4 Sample Collection

Mice were euthanized by cardiac puncture under anesthesia (ketamine 50 mg/kg + xylazine 20 mg/kg). Subcutaneous and epididymal adipose tissues were collected, weighed, and processed.

### 2.5 Histological Analysis and Adipocyte Morphometry

Histological analyses were performed as described by de Assis‐Ferreira et al. (2021) Briefly, adipose samples were fixed in 10% paraformaldehyde in 100 mM phosphate buffer (pH 7.4) for 30 min, cryoprotected in 10% paraformaldehyde with 10% sucrose, then in 20% sucrose at 4°C overnight and embedded in paraffin. Five‐micrometer sections were stained with hematoxylin and eosin (H&E) and analyzed at ×200 magnification. Adipocyte diameter was measured using ImageJ software (NIH, USA) and volume was calculated assuming spherical morphology using: 𝑉 = 4/3𝜋𝑟^3^, where *r* represents the adipocyte radius (diameter/2).

### 2.6 Conditioned Medium Preparation

SAT and EAT fragments (50 mg) were rinsed, cleaned with PBS, and incubated in 500 μL of DMEM/M199 (1:1; Sigma Aldrich) at 37°C and 5% CO_2_ for 24 h. Conditioned media were collected on ice, centrifuged (350 × *g*, 10 min, 4°C), and stored at −80°C. Intact culture medium (DMEM/M199 1:1 incubated under identical conditions without tissue) was processed and analyzed in parallel as the baseline reference. Metabolite levels in conditioned media were interpreted relative to intact medium: values above were classified as net secretion, whereas values below were classified as net uptake.

### 2.7 NMR‐Based Exometabolomics

Frozen supernatants were thawed and mixed with D2O (10% final concentration, v/v) and transferred to a 5 mm NMR tube. Spectra were acquired in a 500.13 MHz Bruker magnet equipped with a TXI probe and an automated sample changer. Data were collected using the zgesgp pulse program, at 298 K with 64 K points, 512 scans, a 20 ppm spectral width, 3.27 s acquisition time, a transmitter offset of 4.704 ppm and baseopt digitalization. Spectra were processed in MestReNova (v14.2.1‐27684) using manual phase correction, baseline correction and chemical‐shift calibration and then binned at 0.02 ppm using average‐sum method. Binned data were normalized to the total spectral intensity (sum normalization) and regions corresponding to water, ethanol and HEPES were excluded. After preprocessing, the data matrix comprised 209 bins (“buckets”) across 23 samples. Metabolite assignments were performed using ^1^H‐^13^C‐HSQC‐based matching in COLMARm (Bingol et al. 2016) with default parameters and confirmed by overlay of 1D ^1^H resonances with spectra from the Biological Magnetic Resonance Data Bank (BMRB) and HMDB 5.0 (Hoch et al. 2023; Wishart et al. 2022). After confirming assignments, nonoverlapping buckets corresponding to diagnostic chemical‐shift regions were selected for metabolite‐specific quantification in univariate analyses. Metabolite signal intensities were therefore extracted from the normalized bucket values rather than by peak picking.

### 2.8 RNA Extraction and Quantitative Real‐Time PCR

Total RNA from SAT and EAT was extracted using PureLink RNA Mini Kit (Invitrogen). DNAse treatment (Promega) preceded cDNA synthesis with the Go Taq Probe 2‐Step RT‐qPCR System (Promega). qPCR was performed with SYBR Green on a Rotor‐Gene Q (Qiagen). Gene expression was normalized to 18S rRNA using the 2^−ΔΔCt^ method. Primer sequences are provided in Table S3.

### 2.9 Lactate Quantification

Serum and conditioned‐medium lactate levels were measured using an enzymatic kit (Bioclin K084, Brazil). The assay is based on LDH‐mediated oxidation of l‐lactate to pyruvate and NAD^+^ reduction to NADH, monitored at 340 nm. Lactate concentrations were calculated using the standard curve and expressed as mg/dL.

### 2.10 Statistical Analysis

The binned and sum‐normalized NMR dataset was analyzed in Metaboanalyst 5.0 for Principal Component Analysis (PCA) and in GraphPad Prism version 9.5.1 for normality testing and univariate statistics. For univariate analysis of spectral bins/metabolites, pairwise comparisons between independent experimental groups were performed using the Mann–Whitney *U* test, with adjustment for multiple testing by controlling the false discovery rate (FDR) using the two‐stage step‐up procedure of Benjamini, Krieger, and Yekutieli (*q* = 0.05). Paired comparisons between SAT and EAT explants obtained from the same animal were performed using the Wilcoxon matched‐pairs signed‐rank test with the same FDR approach. Associations between variables were assessed using Spearman's rank correlation. For nonmetabolomic endpoints, data normality was assessed Shapiro Wilk test. Longitudinal/time course data (e.g., ipGTT glycemic curve) were analyzed using two‐way repeated measures ANOVA (time × group), followed Tukey's multiple‐comparisons test. Single time‐point outcomes (e.g., tissue mass, adipocyte volume, fasting glucose, ipGTT AUC, gene expression, and lactate concentrations) were analyzed by one‐way ANOVA followed by Tukey's multiple‐comparisons test when normally distributed or by Kruskal–Wallis test followed by Dunn's multiple‐comparisons test when normality assumptions were not met. Data are presented as mean ± SEM. A two‐sided *p* < 0.05 was considered statistically significant and for metabolomics univariate analyses significance was additionally interpreted under FDR control (*q* < 0.05).

## 3 Results

### 3.1 Effect of Chia Oil Supplementation on Body Mass, Adipose Tissue Mass, Adipocyte Hypertrophy and Glucose Homeostasis

The experimental design is illustrated in Figure 1A. As expected, HFD feeding significantly increased body mass compared with control mice (Figure 1B). Chia oil supplementation did not alter body mass under either diet (CC or HC groups). SAT and EAT masses were significantly higher in obese mice compared to control mice (Figure 1C,D) and chia oil supplementation did not significantly change the mass of either adipose tissue depot in control or obese animals.

**FIGURE 1:**
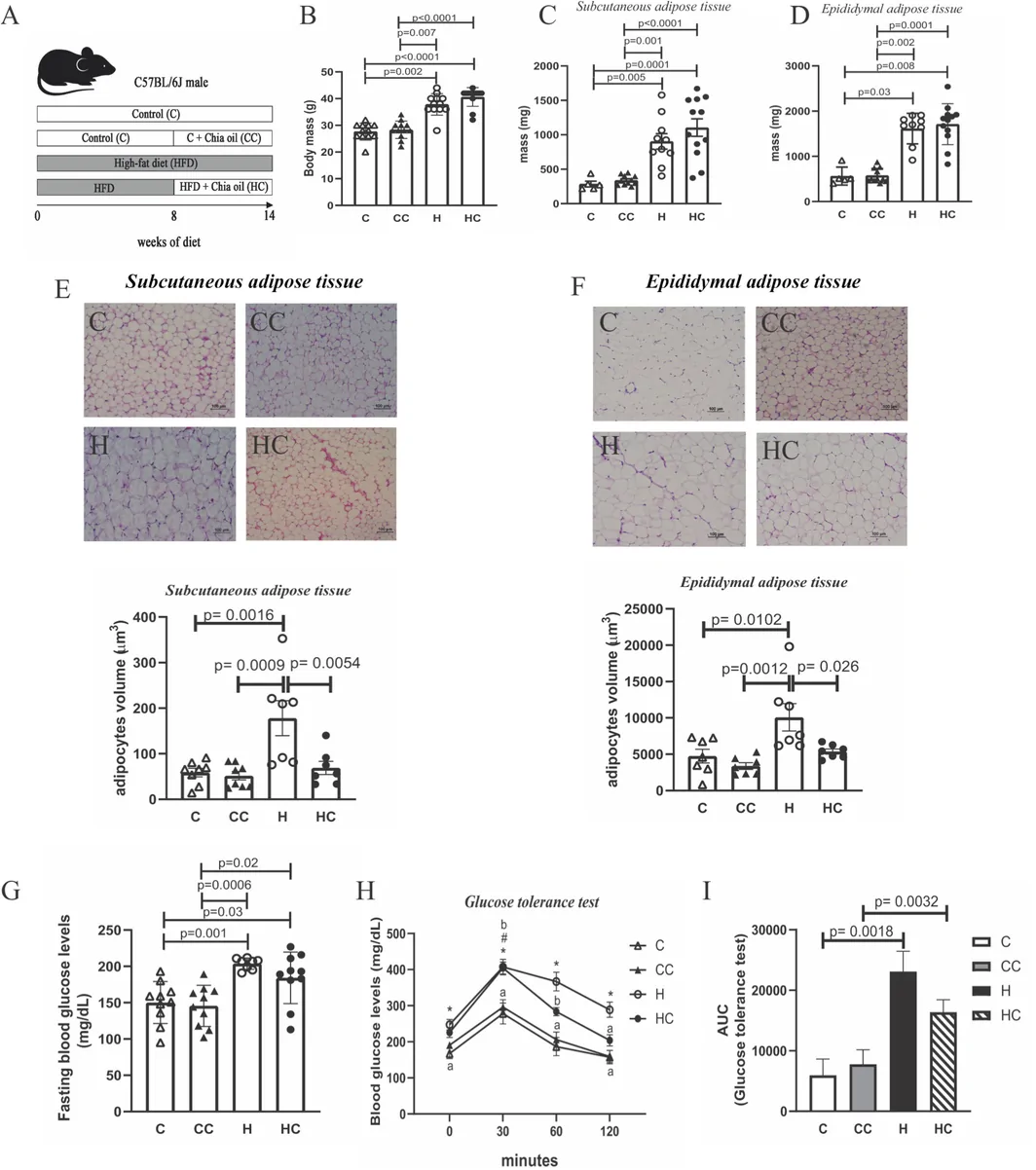
Effects of a high‐fat diet and chia supplementation (A) on body mass (B), SAT mass (C), EAT mass (D), histology and morphometry of SAT (E) and EAT (F), fasting glucose levels (G), intraperitoneal glucose tolerance test (ipGTT) (H) and area under the curve of ipGTT (I) in male mice. C: mice fed with control diet; CC: mice fed with control diet supplemented with seed chia oil (1.5% (w/w)); H: mice fed with a high‐fat diet; HC: mice fed with a high‐fat diet supplemented with chia oil (1.5% (w/w)). Data are presented as means ± SEM. *n* = 5–12 animals per group. *p* values are indicated in the figure. *p* values are indicated in the figures.

Adipocyte hypertrophy was evident in both SAT and EAT of obese mice (Figure 1E,F, respectively). Notably, chia oil supplementation significantly reduced adipocyte volume in both depots of obese mice (Figure 1E,F), indicating attenuation of obesity‐associated hypertrophy despite unchanged depot mass.

Fasting glucose was elevated in obese mice (Figure 1G). During the ipGTT, obese mice exhibited glucose intolerance (Figure 1H). Chia oil supplementation lowered the glycemic curve across time points and reduced AUC, particularly in obese mice (Figure 1I), indicating improved whole‐body glucose handling.

### 3.2 Effects of Chia Oil Supplementation on Adipose Tissue Metabolic Profiles

NMR‐based exometabolomics identified 29 metabolites in conditioned medium from SAT and EAT explants (Table S4 and Figure S1). PCA demonstrated that chia oil supplementation affected the global metabolic profile of both depots (Figure 2A,B). SAT showed modest group separation, whereas EAT displayed a clearer clustering pattern, indicating greater metabolic responsiveness. EAT from chia oil‐supplemented obese mice (HC) shifted toward those from chia‐supplemented controls (CC), suggesting partial metabolic normalization.

**FIGURE 2:**
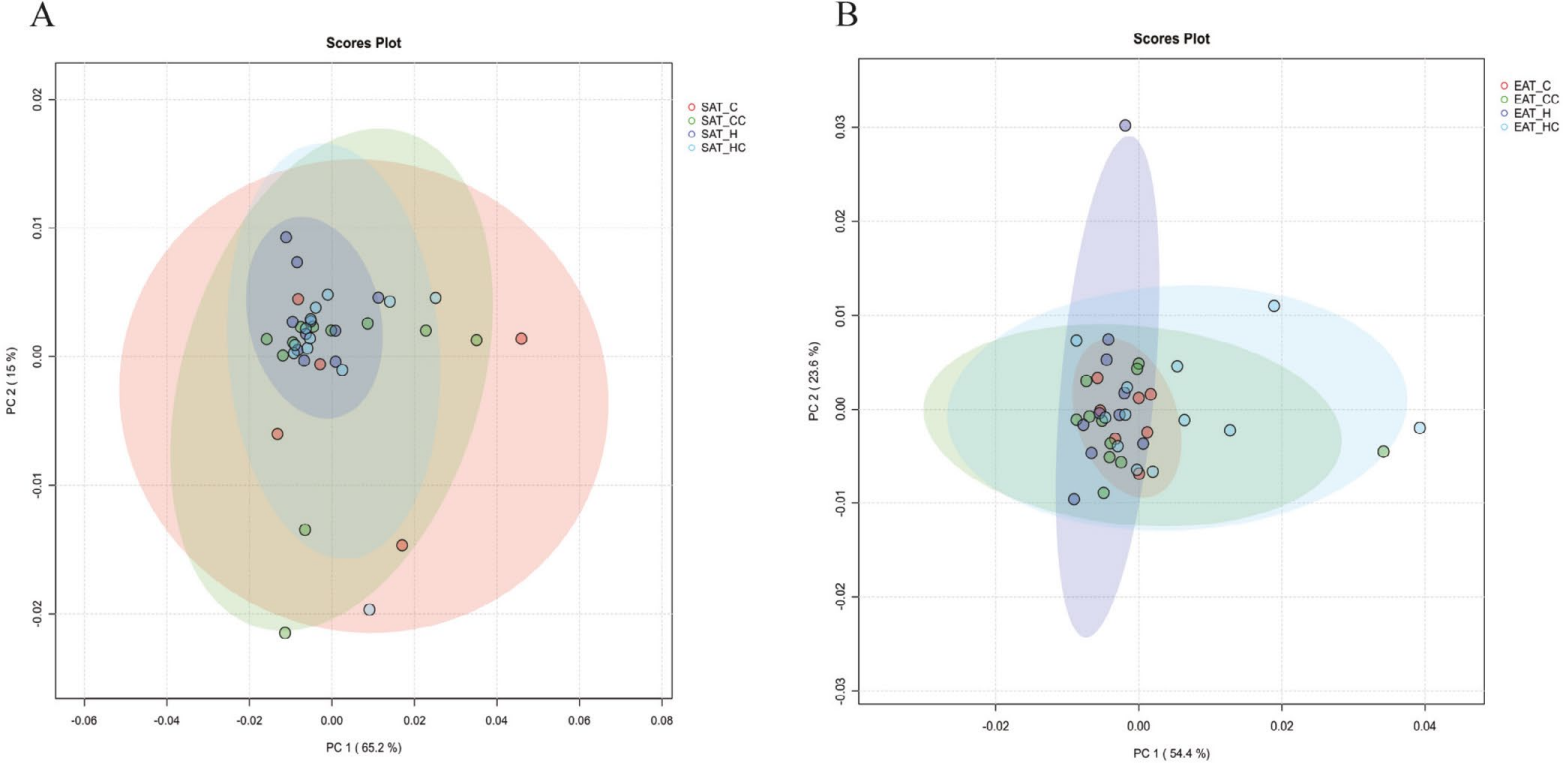
Effects of chia oil supplementation on the metabolic profile of subcutaneous (SAT) (A) and epididymal adipose tissues (EAT) (B) in male mice. Principal component analysis (PCA) scores plot indicates detectable changes in the exportation and uptake of metabolites in both SAT and EAT.

### 3.3 Obesity and Chia Oil Supplementation Induced Changes in Metabolites Levels in Conditioned Medium of Subcutaneous and Epididymal Adipose Tissue Explants

Quantitative analysis of SAT‐conditioned media revealed several statistical changes associated with obesity and chia oil supplementation (Table 1; Figure 3; Figure S2). Tables 1 and 2 summarize all assigned metabolites, fold‐changes, and *p*‐values for SAT and EAT, respectively. Among all metabolites detected, histidine, valine, proline, glutamate, and threonine showed the most pronounced differences. Obesity alone reduced histidine levels in the conditioned medium of SAT (H vs. C, *p* = 0.004), and chia oil further amplified this effect in the control group (CC vs. C, *p* = 0.03). Additionally, chia oil supplementation in obese mice decreased SAT release of BCAA valine (HC vs. H, *p* = 0.04), consistent with altered BCAA metabolism. Two amino acids linked to extracellular matrix remodeling, proline and glutamate, were also significantly reduced by chia oil in obese SAT (*p* = 0.03 for both). Conversely, threonine secretion increased in HC mice (*p* = 0.04), indicating shifts in nonessential amino acid handling.

**TABLE 1:** Comparison of normalized median intensities and fold changes of metabolites in SAT.

^1^H chemical shift, ppm	Metabolite	C	CC	H	HC	CC:C (fold change)	CC:C (p value)	H:C (fold change)	H:C (p value)	HC:H (fold change)	HC:H (p value)
0.88, 1.69	Ethylmalonate	6.51E−04	4.17E−04	4.93E−04	4.75E−04	6.41E−01	7.64E−01	7.57E−01	7.68E−01	9.63E−01	7.09E−01
0.92, 0.93, 0.99, 1.25, 1.96, 1.46, 3.66	l‐Isoleucine	2.72E−03	2.45E−03	2.74E−03	2.52E−03	9.01E−01	2.12E−01	1.01E+00	7.00E−01	9.18E−01	1.75E−01
0.94, 0.95, 1.6, 3.72	Leucine	1.06E−02	9.82E−03	1.07E−02	1.04E−02	9.26E−01	6.42E−01	1.01E+00	6.58E−01	9.72E−01	4.26E−01
0.9, 1.03, 2.25, 3.60	l‐Valine	8.82E−03	8.68E−03	8.96E−03	8.54E−03	9.84E−01	3.63E−01	1.02E+00	7.92E−01	9.53E−01	**4.07E−02**
1.31, 3.56, 4.24^a^	l‐Threonine	1.06E−03	1.10E−03	1.07E−03	1.19E−03	1.04E+00	9.76E−01	1.01E+00	5.73E−01	1.11E+00	**4.75E−02**
1.32, 4.10	Lactate	1.50E−02	1.47E−02	1.38E−02	1.55E−02	9.80E−01	5.10E−01	9.20E−01	2.16E−01	1.12E+00	6.64E−02
1.46, 3.76	Alanine	3.95E−03	3.84E−03	4.00E−03	3.98E−03	9.72E−01	3.91E−01	1.01E+00	8.59E−01	9.95E−01	9.36E−01
1.64, 1.71, 1.88, 3.23	l‐Arginine	1.33E−03	1.14E−03	1.18E−03	1.15E−03	8.57E−01	2.33E−01	8.87E−01	6.56E−01	9.75E−01	7.57E−02
1.92	Acetate	6.58E−03	6.28E−03	7.02E−03	6.98E−03	9.54E−01	> 0.99	1.07E+00	4.57E−01	9.94E−01	5.39E−01
1.98, 2.35, 4.14	l‐Proline	1.54E−03	1.35E−03	1.48E−03	1.32E−03	8.77E−01	9.52E−02	9.58E−01	7.93E−01	8.95E−01	**3.11E−02**
2.08, 2.33, 3.75	l‐Glutamate	2.03E−03	1.73E−03	1.92E−03	1.80E−03	8.52E−01	2.21E−01	9.43E−01	7.89E−01	9.40E−01	**3.13E−02**
2.14, 2.43, 3.75	l‐Glutamine	1.24E−02	1.30E−02	1.21E−02	1.23E−02	1.05E+00	3.07E−01	9.76E−01	3.88E−01	1.01E+00	3.05E−01
3.28, 3.27, 3.97, 3.09, 7.31	l‐Phenylanaline	3.88E−03	4.11E−03	4.42E−03	4.09E−03	1.06E+00	3.62E−01	1.14E+00	4.40E−01	9.25E−01	8.59E−01
3.56, 3.75, 3.65	Glycerol	1.51E−02	1.44E−02	1.49E−02	1.48E−02	9.54E−01	3.77E−01	9.83E−01	7.44E−01	9.97E−01	6.16E−01
3.23, 3.40, 3.53, 3.69, 3.71, 3.76, 3.81, 3.87, 5.22^a^	d‐Glucose	2.06E−03	2.33E−03	2.07E−03	2.30E−03	1.13E+00	3.91E−01	1.00E+00	9.53E−01	1.11E+00	6.69E−02
7.11, 7.20, 7.26^a^, 7.28, 7.40, 7.72	l‐Tryptophan	2.77E−04	2.42E−04	2.41E−04	2.40E−04	8.74E−01	5.10E−01	8.70E−01	4.96E−01	9.96E−01	4.96E−01
3.03, 3.17, 3.91, 3.93, 6.86^a^, 7.17	l‐Tyrosine	9.33E−04	1.00E−03	8.50E−04	9.10E−04	1.07E+00	6.81E−01	9.11E−01	3.71E−01	1.07E+00	2.75E−01
3.07, 3.94, 7.04^a^, 8.03	l‐Histidine	9.78E−04	9.05E−04	9.04E−04	9.14E−04	9.25E−01	**3.98E−02**	9.24E−01	**4.33E−03**	1.01E+00	1.97E−01
8.46	Formate	5.63E−05	4.89E−05	5.61E−05	5.78E−05	8.69E−01	7.43E−01	9.96E−01	9.53E−01	1.03E+00	8.21E−01
7.58, 8.23, 8.68, 8.70^a^	Nicotinamide	5.79E−05	4.60E−05	6.12E−05	4.67E−05	7.94E−01	1.21E−01	1.06E+00	7.68E−01	7.63E−01	1.07E−01

*Note:* The bold values represent the significant *p*‐values.

^a^ Nonoverlapping peaks selected for relative quantification.

**FIGURE 3:**
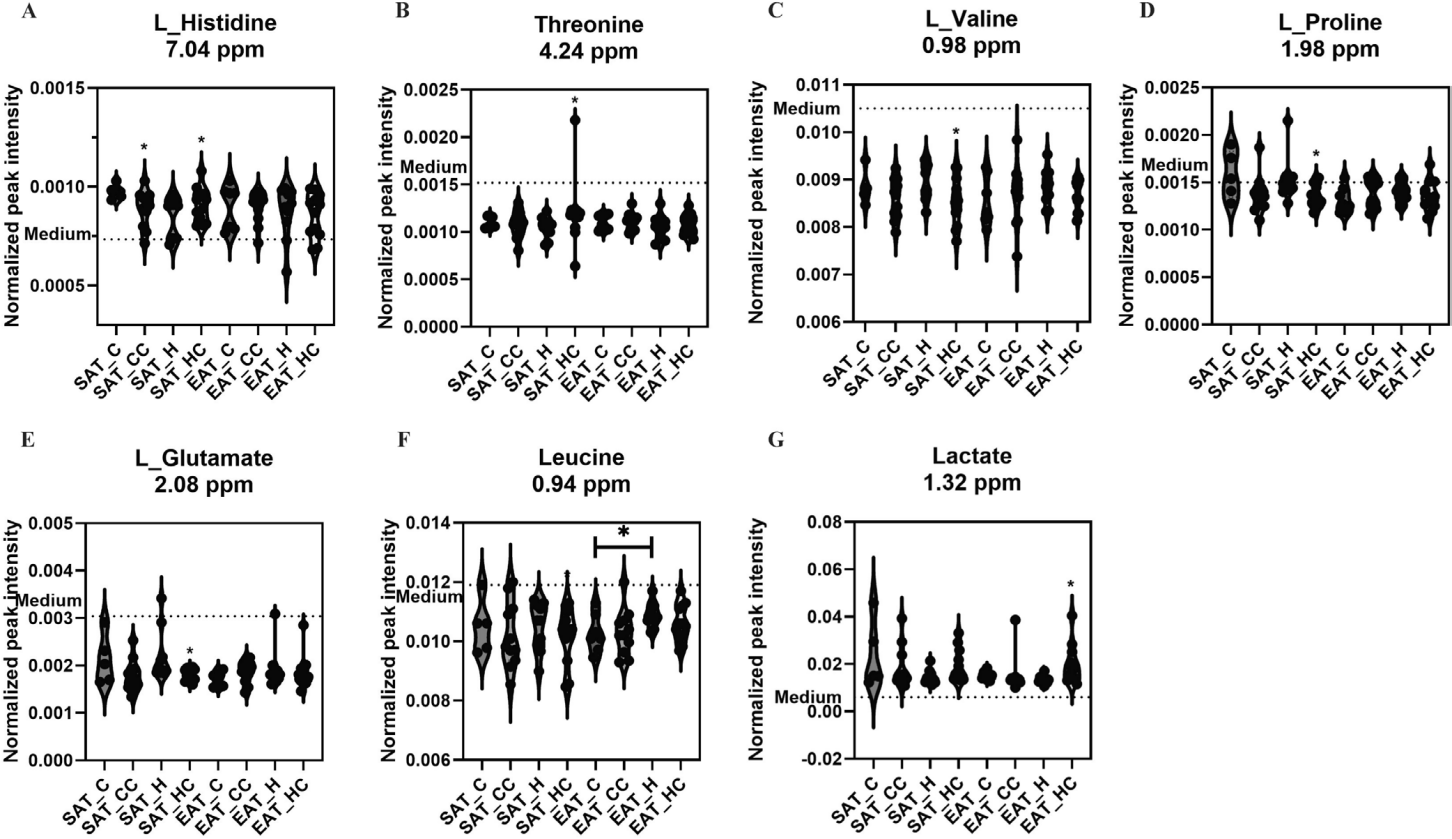
Normalized peak intensities of selected metabolites in subcutaneous (SAT) and epididymal adipose tissue (EAT). Control diet (C), chia‐supplemented control diet (CC), high‐fat diet (H), and chia‐supplemented high‐fat diet (HC). The violin plots show changes in the levels of (A) l‐Histidine (7.04 ppm), (B) threonine (4.24 ppm), (C) l‐Valine (0.98 ppm), (D) l‐Proline (1.98 ppm), (E) L‐Glutamate (2.08 ppm), (F) Leucine (0.94 ppm), (G) Lactate (1.32 ppm). The dotted line indicates the presence (normalized peak intensity) of the metabolite in the intact culture medium. Significant differences were indicated by asterisks (*p < 0.05).

**TABLE 2:** Comparison of normalized median intensities and fold changes of metabolites in EAT.

^1^H chemical shift, ppm	Metabolite	C	CC	H	HC	CC:C (fold change)	CC:C (*p* value)	H:C (fold change)	H:C (*p* value)	HC:H (fold change)	HC:H (*p* value)
0.88, 1.69	Ethylmalonate	3.33E−04	3.69E−04	4.22E−04	3.52E−04	1.1E+00	3.7E−01	1.3E+00	2.4E−01	8.3E−01	2.0E−01
0.92, 0.93, 0.99, 1.25, 1.96, 1.46, 3.66	l‐Isoleucine	2.35E−03	2.55E−03	2.68E−03	2.65E−03	1.1E+00	2.7E−01	1.1E+00	7.4E−02	9.9E−01	9.1E−01
0.94, 0.95, 1.69, 3.72	Leucine	1.01E−02	1.02E−02	1.09E−02	1.06E−02	1.0E+00	> 0.99	1.1E+00	**4.5E−02**	9.7E−01	2.0E−01
0.98, 1.03, 2.25, 3.60	l‐Valine	8.31E−03	8.77E−03	8.89E−03	8.75E−03	1.1E+00	7.2E−01	1.1E+00	2.3E−01	9.8E−01	3.2E−01
1.31, 3.56, 4.24^a^	l‐Threonine	1.08E−03	1.12E−03	1.06E−03	1.09E−03	1.0E+00	9.1E−01	9.8E−01	5.2E−01	1.0E+00	6.2E−01
1.32, 4.10	Lactate	1.53E−02	1.36E−02	1.37E−02	1.83E−02	8.9E−01	5.5E−02	9.0E−01	6.6E−02	1.3E+00	**2.0E−02**
1.46, 3.76	Alanine	3.69E−03	3.80E−03	3.77E−03	3.70E−03	1.0E+00	7.7E−01	1.0E+00	7.6E−01	9.8E−01	9.4E−01
1.64, 1.71, 1.88, 3.23	l‐Arginine	1.06E−03	1.16E−03	1.12E−03	1.01E−03	1.1E+00	5.5E−01	1.1E+00	3.0E−01	9.0E−01	6.1E−02
1.92	Acetate	7.68E−03	7.03E−03	7.21E−03	7.72E−03	9.2E−01	1.2E−01	9.4E−01	9.2E−02	1.1E+00	1.2E−01
1.98, 2.35, 4.14	l‐Proline	1.27E−03	1.45E−03	1.42E−03	1.35E−03	1.1E+00	2.8E−01	1.1E+00	2.2E−01	9.5E−01	4.3E−01
2.08, 2.33, 3.75	l‐Glutamate	1.71E−03	1.88E−03	1.81E−03	1.76E−03	1.1E+00	1.3E−01	1.1E+00	1.5E−01	9.7E−01	5.7E−01
2.14, 2.43, 3.75	l‐Glutamine	1.16E−02	1.17E−02	1.15E−02	1.18E−02	1.0E+00	5.2E−01	9.9E−01	7.2E−01	1.0E+00	8.8E−01
3.28, 3.27, 3.97, 3.09, 7.31	l‐Phenylanaline	3.97E−03	4.38E−03	3.96E−03	4.08E−03	1.1E+00	4.6E−01	1.0E+00	9.1E−01	1.0E+00	5.0E−01
3.56, 3.75, 3.65	Glycerol	1.43E−02	1.44E−02	1.35E−02	1.48E−02	1.0E+00	9.8E−01	9.4E−01	1.3E−01	1.1E+00	1.3E−01
3.23, 3.40, 3.53, 3.69, 3.71, 3.76, 3.81, 3.87, 5.22^a^	d‐Glucose	2.29E−03	2.33E−03	2.31E−03	2.33E−03	1.0E+00	8.4E−01	1.0E+00	4.3E−01	1.0E+00	8.8E−01
7.11, 7.20, 7.26^a^, 7.28, 7.40, 7.72	l‐Tryptophan	2.36E−04	2.42E−04	2.25E−04	2.23E−04	1.0E+00	9.1E−01	9.5E−01	2.3E−01	9.9E−01	8.1E−01
3.03, 3.17, 3.91, 3.93, 6.86^a^, 7.17	l‐Tyrosine	9.03E−04	8.98E−04	8.30E−04	8.28E−04	9.9E−01	9.8E−01	9.2E−01	1.7E−01	1.0E+00	5.8E−01
3.07, 3.94, 7.04^a^, 8.03	l‐Histidine	9.67E−04	9.29E−04	9.07E−04	8.90E−04	9.6E−01	6.4E−01	9.4E−01	8.9E−01	9.8E−01	6.3E−01
8,46	Formate	4.59E−05	4.92E−05	4.83E−05	5.61E−05	1.1E+00	8.8E−01	1.1E+00	8.1E−01	1.2E+00	8.2E−01
7.58, 8.23, 8.68, 8.70^a^	Nicotinamide	4.99E−05	6.14E−05	6.48E−05	4.65E−05	1.2E+00	1.4E−01	1.3E+00	6.7E−01	7.2E−01	4.6E−01

*Note:* The bold values represent the significant *p*‐values.

^a^ Nonoverlapping peaks selected for relative quantification.

Metabolite profiling of EAT revealed a pattern of metabolic changes distinct from SAT (Table 2; Figure 3; Figure S2). Obesity increased the secretion of the BCAA leucine (H vs. C; *p* = 0.04). Chia oil did not significantly alter leucine secretion in lean or obese animals. The metabolite most strongly influenced by chia oil in EAT was lactate. Chia oil markedly increased lactate levels in conditioned medium from EAT of obese mice (HC vs. H, *p* = 0.02), suggesting enhanced glycolytic flux or depot‐specific metabolic adaptation. Other amino acids, including isoleucine, valine, proline, glutamate, glutamine, alanine, and phenylalanine showed modest obesity—or supplementation—related shifts, but none reached statistical significance.

To functionally validate the metabolomic finding suggesting altered glycolytic flux in EAT, we quantified lactate levels in serum and in conditioned media from adipose tissue explants. Despite the absence of significant differences in serum lactate (Figure S3), explants from EAT of the HC group released significantly more lactate than those from H group (HC vs. H, *p* = 0.04) supporting the notion that chia oil supplementation locally alters glycolytic output in EAT without producing systemic alterations. In SAT, lactate levels were significantly higher in C group relative to CC (*p* = 0.004) and H (*p* = 0.002), reinforcing a depot‐specific modulation of lactate metabolism (Figure S3).

Consistent with these biochemical findings, gene expression analysis of monocarboxylate transporters (MCT1 and MCT4) supports a divergent regulation of lactate handling between depots. In SAT, MCT4 expression was upregulated in the CC group compared to C, H and HC groups (*p* = 0.04; 0.01 and 0.01, respectively). In contrast, MCT1 expression remained unchanged across all SAT groups (Figure S3). No significant alterations in the expression of either MCT1 or MCT4 were detected in EAT across groups, indicating that the enhanced lactate release observed in this depot in the HC group is not driven by transcriptional regulation of these transporters.

Overall, Figure 3 illustrates these univariate differences, showing that EAT exhibits a stronger glycolytic signature (via lactate) in response to chia oil, whereas SAT shows greater modulation of amino acid pathways, especially those linked to ECM turnover and BCAA metabolism.

### 3.4 Acetate Secretion Differs Between SAT and EAT Only in the HC Group

Analysis of acetate (1.92 ppm) revealed no significant differences among dietary groups within each adipose depot (Figure 4). In both SAT and EAT, acetate release remained similar across C, CC, H, and HC mice. However, a clear depot‐specific difference emerged exclusively in the HC group, in which EAT released significantly more acetate than SAT. No SAT–EAT differences were detected in any of the other groups. These findings indicate that acetate modulation is not driven by obesity or chia oil treatment per se but reflects a depot‐dependent metabolic response restricted to the HC condition.

**FIGURE 4:**
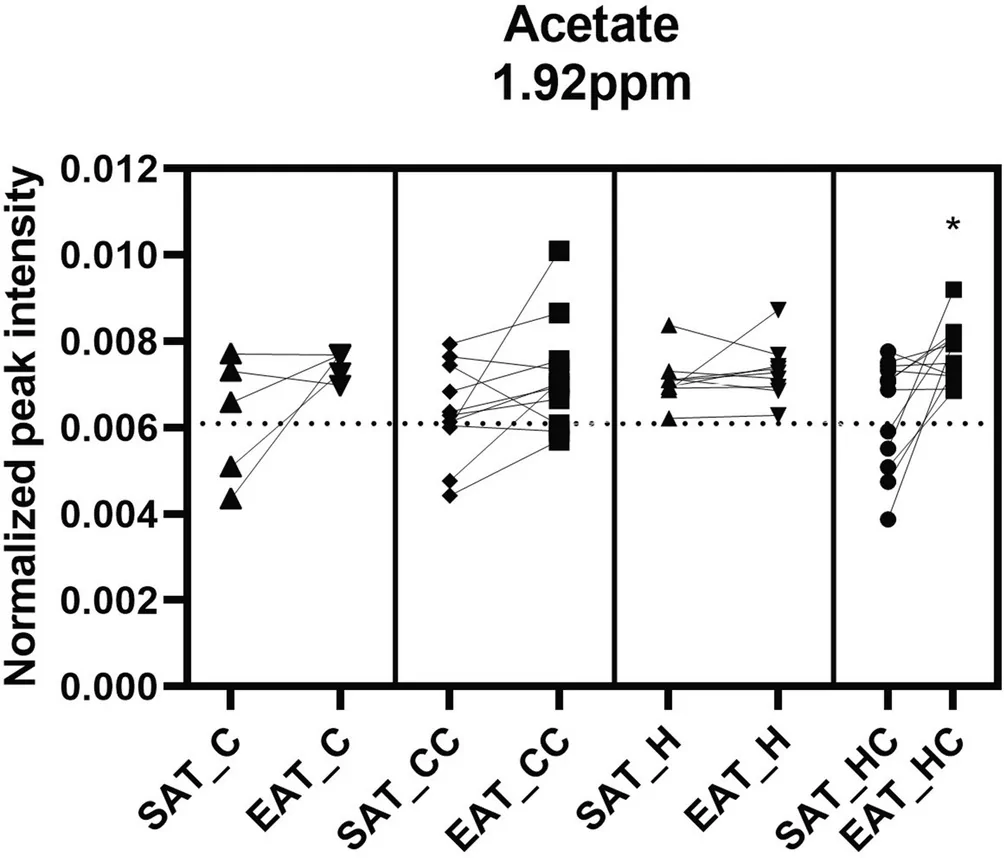
Acetate was the most altered metabolite between paired SATs and EATs from the same mice. Control diet (C), chia‐supplemented control diet (CC), high‐fat diet (H), and chia‐supplemented high‐fat diet (HC). Each symbol represents individual mice, and lines connect the same animals across different conditions. The dotted line indicates the presence (normalized peak intensity) of the metabolite in the intact culture medium. Significant differences were indicated by the asterisk (*p < 0.05).

### 3.5 Chia Oil Supplementation Reshapes the Metabolic Correlation Networks in SAT and EAT

To assess how chia oil supplementation modifies the coordination between secreted metabolites, Spearman correlation matrices were generated for SAT and EAT explants under control and obese conditions (Figure 5, Figure S4). This analysis allowed us to map metabolic relationships beyond individual metabolite changes.

**FIGURE 5:**
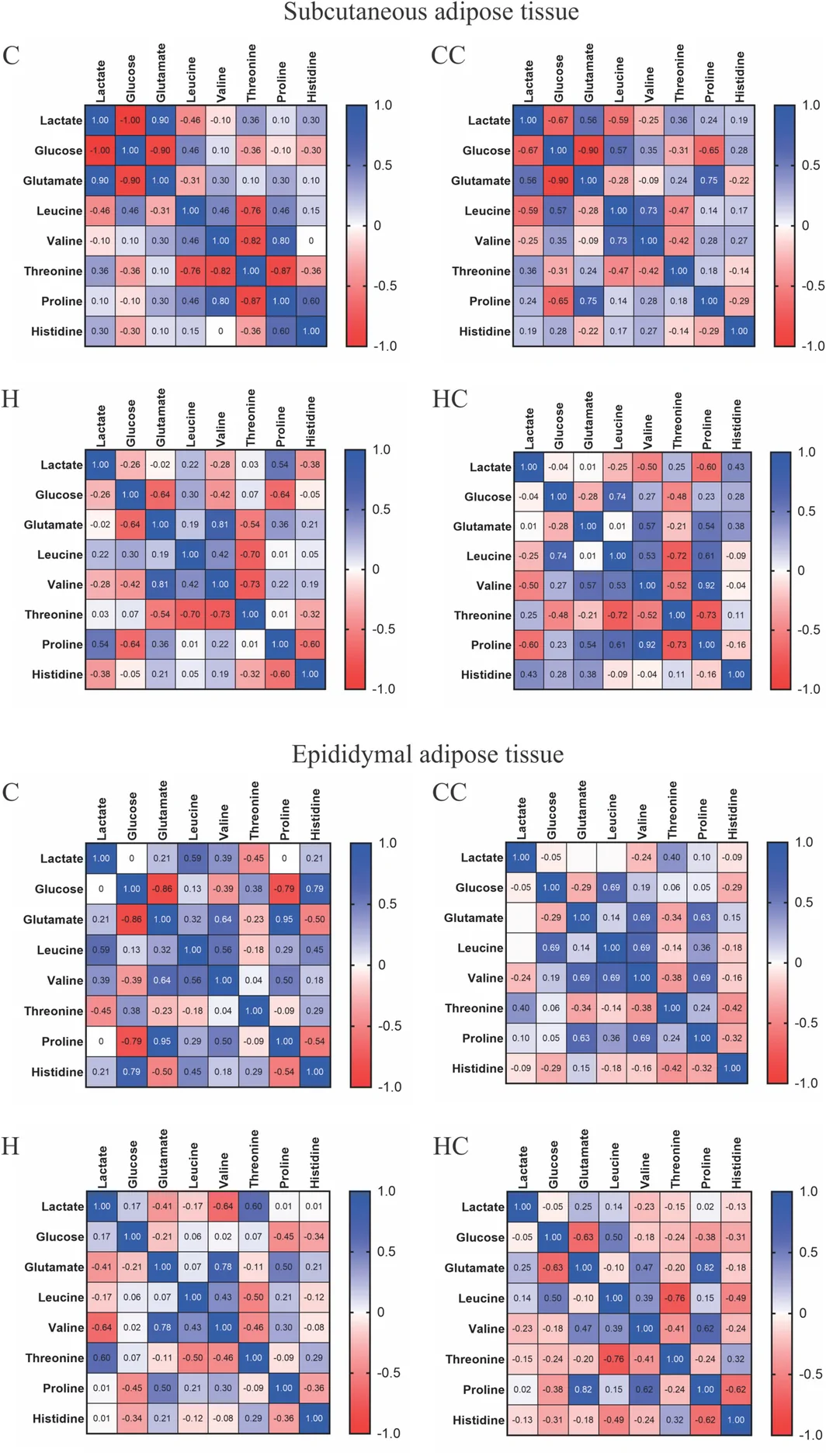
Chia oil supplementation differentially remodels the correlation networks of metabolites secreted by SAT and EAT explants. Spearman correlation heatmaps illustrate the relationships among selected metabolites in conditioned media from SAT (A–D) and EAT (E–H) explants under control diet (C), control+chia oil (CC), high‐fat diet (H), and high‐fat diet+chia oil (HC). Correlations are based on most significant assigned buckets described in Tables 1 and 2 for each condition. Lactate (1.32 ppm), glucose (5.22 ppm), l‐glutamate (2.08 ppm), leucine (0.94 ppm), l‐valine (0.98 ppm), threonine (4.24 ppm), l‐proline (1.98 ppm), and l‐histidine (7.04 ppm). Corresponding *p* values are provided in Figure S4.

In SAT from control mice (Figure 5A), we identified the expected strong negative correlation between glucose and lactate (*r* = −1.0, *p* = 0.016), consistent with canonical glycolytic flux. Chia oil supplementation weakened this relationship (*r* = −0.67, *p* = 0.027; Figure 5B), suggesting a redistribution of glucose carbon toward alternative pathways. In CC mice, chia oil also introduced new correlations, including a robust negative glucose‐glutamate correlation (*r* = −0.9, *p* = 0.0004) and negative glucose‐proline association (*r* = −0.65, *p* = 0.033), as well as positive correlations between proline‐glutamate (*r* = 0.75, *p* = 0.009) and valine–leucine (*r* = 0.73; *p* = 0.013), indicating that amino‐acid handling becomes more tightly interlinked.

In obese mice (Figure 5C), SAT displayed a negative glucose‐glutamate correlation (*r* = −0.64, *p* = 0.049) and a strong positive glutamate‐valine association (*r* = 0.81, *p* = 0.006). Chia oil supplementation disrupted several of these relationships (Figure 5D); the glucose‐glutamate correlation was attenuated (*r* = −0.28, *p* = 0.381) and the glutamate‐valine correlation weakened (*r* = 0.57, *p* = 0.055). Conversely, chia oil induced new correlations in obese SAT, including positive associations between glucose‐leucine (*r* = 0.74, *p* = 0.007), proline–leucine (*r* = 0.61; *p* = 0.040) and proline–valine (*r* = 0.92, *p* = 0.0001), as well as negative correlations between lactate‐proline (*r* = −0.60, *p* = 0.042) and threonine‐proline (*r* = −0.73, *p* = 0.008). These changes collectively show that chia oil reconfigures SAT amino‐acid relationships, weakening classical glycolytic associations and strengthening connections between BCAAs, proline, and glutamate.

In EAT of control mice (Figure 5E), glucose showed strong negative correlations with glutamate (*r* = −0.86, *p* = 0.023) and proline (*r* = −0.79, *p* = 0.041) and a positive correlation with histidine (*r* = 0.79, *p* = 0.048). These glucose‐centered relationships were largely lost following chia oil supplementation (Figure 5F), indicating a reorganization of substrate processing. Chia oil introduced new positive correlations in CC mice, including glucose–leucine, glutamate–valine, leucine–valine, and proline–valine (all *r* = 0.69, *p* = 0.022), suggesting tighter integration of BCAA and proline metabolism.

In obese EAT (Figure 5G), the dominant association was a positive correlation between glutamate and valine (*r* = 0.78, *p* = 0.009). Chia oil supplementation profoundly reshaped these networks (Figure 5H), generating new negative correlations, including glucose‐glutamate (*r* = −0.63, *p* = 0.031), leucine‐threonine (*r* = −0,76, *p* = 0.005) and histidine‐proline (*r* = −0.62, *p* = 0.036). Additionally, chia oil strengthened positive correlations between glutamate‐proline (*r* = 0.82, *p* = 0.001) and proline valine (*r* = 0.62, *p* = 0.035). Together, these results show that chia oil induces a broad reorganization of metabolic networks in EAT, shifting relationships among glucose, amino acids and glycolytic products.

## 4 Discussion

In this study, we employed NMR‐based exometabolomics to characterize metabolic secretion and uptake patterns in conditioned medium from subcutaneous (SAT) and epididymal (EAT) adipose tissue explants of obese and control mice supplemented with chia oil. Our findings show that chia oil supplementation promotes coordinated metabolic remodeling of adipose tissue in obese mice, despite unchanged body weight or adipose tissue mass. Chia oil reduced adipocyte hypertrophy and improved glucose tolerance in obese mice, supporting previous evidence of its role in enhancing insulin sensitivity and modulating body composition in obesity (de Souza et al. 2020; Fonte‐Faria et al. 2019). In this study, these physiological improvements contextualize the depot‐specific metabolic adaptations revealed by the exometabolomic analyses.

Chia oil was evaluated here as a dietary supplementation intervention incorporated into the base diets at 1.5% (w/w). Accordingly, the supplemented diets differ from their respective controls by the addition of chia oil, resulting in a modest increase in total lipids and energy density while producing a marked shift in fatty‐acid quality, with increased α‐linolenic acid and a lower ω‐6:ω‐3 ratio. Thus, we interpret the present results as the metabolic effects of chia oil supplementation within this defined diet formulation, consistent with prior work using dietary chia oil supplementation protocols (de Assis‐Ferreira et al. 2026).

Obesity markedly reduced histidine secretion from SAT, a pattern that likely reflects impaired amino‐acid management rather than a favorable metabolic adaptation, given the well‐described insulin‐sensitizing and anti‐inflammatory actions of histidine (Sun et al. 2014). Chia oil supplementation further reduced histidine secretion only in the control mice, indicating that chia oil can modulate histidine metabolism under physiological conditions but does not override the suppressive effects of obesity. Because lower extracellular histidine levels do not necessarily equate to reduced availability—histidine may be retained intracellularly or redirected toward antioxidant and metabolic pathways—the functional implications of this change remain uncertain. Importantly, chia oil did not exacerbate the obesity‐induced reduction in histidine secretion, suggesting that histidine metabolism in SAT is already constrained under obesogenic stress. Overall, these findings indicate that histidine dynamics in SAT reflect underlying metabolic stress in obesity and modest nutritional modulation in lean animals, rather than representing a direct mechanism of improved insulin sensitivity or anti‐inflammatory action in response to chia supplementation.

Chia oil supplementation also influenced branched‐chain amino acid (BCAA) metabolism in a depot‐specific manner. In SAT, chia oil reduced l‐valine secretion in obese mice, a finding that aligns with clinical and experimental evidence linking elevated circulating BCAAs to insulin resistance (Thaker et al. 2024). This decrease may therefore reflect improved adipocyte metabolic handling and reduced reliance on BCAA catabolism as an anaplerotic fuel source. However, this effect was not uniformly observed across depots: despite obesity‐induced increases in l‐leucine secretion in EAT, chia oil did not modify leucine levels in this visceral depot. This divergence suggests that the impact of chia oil on BCAA metabolism depends on the intrinsic metabolic characteristics of each fat depot. Whereas SAT appears more responsive to chia‐induced adjustments in amino‐acid trafficking, EAT may exhibit a more rigid BCAA profile under obesogenic conditions. Taken together, these findings reveal that SAT displays a greater degree of metabolic flexibility in response to chia oil, whereas EAT exhibits more rigid metabolic responses, showing prominent glycolytic shifts but limited modulation of amino acid handling.

Chia oil supplementation also modified amino acids associated with extracellular matrix dynamics and inflammatory–metabolic regulation, although the functional implications of these changes remain uncertain. In SAT from obese mice, chia oil reduced the secretion of both proline and glutamate, two metabolites involved in collagen turnover (Kay et al. 2021) and inflammatory signaling (Chaouche et al. 2024), respectively. While lower extracellular levels could suggest reduced release or increased intracellular retention, these changes cannot be directly interpreted as beneficial or detrimental, as secretion reflects multiple metabolic processes, including synthesis, degradation, and utilization. The concurrent increase in threonine secretion in SAT from obese mice supplemented with chia oil further supports the idea of a shift in amino‐acid routing, possibly reflecting depot‐specific adaptations in energy and nitrogen metabolism (Tang et al. 2021). Rather than indicating improved or impaired ECM or inflammatory status, these alterations likely represent context‐dependent metabolic reorganization of SAT under chia supplementation, consistent with the broader amino‐acid remodeling observed in the exometabolomic analyses. Additional mechanistic studies will be required to determine whether these shifts in amino‐acid handling contribute functionally to adipose tissue structure or metabolic health.

In contrast to the more flexible metabolic adjustments observed in SAT, EAT displayed a distinct pattern characterized primarily by increased lactate secretion in obese mice supplemented with chia oil, which was not associated with changes in the gene expression of MCT1 or MCT4 in this depot. Although chia oil supplementation increased lactate release from EAT explants, this effect did not translate into higher circulating lactate levels, indicating that the observed changes likely reflect local metabolic reprogramming rather than systemic lactate accumulation. Adipose‐derived lactate is rapidly cleared and utilized by neighboring tissues (Wang et al. 2025) and therefore increased lactate flux can occur without altering its steady‐state concentration in plasma. In this context, the greater lactate secretion from EAT of HC group may represent a shift in intracellular carbon handling. While the lactate shuttle provides a conceptual framework for understanding how lactate may act as a metabolic intermediate for other tissues, our findings suggest that chia oil primarily affects local glycolytic activity within EAT, rather than systemic lactate turnover. This depot‐specific adaptation may still contribute to improved adipose tissue function by reducing lipolytic pressure and modifying the metabolic microenvironment of visceral fat.

Although acetate was initially expected to reflect treatment‐induced metabolic modulation, our data indicate a more modest and depot‐specific pattern. Acetate release did not differ among dietary groups within either SAT or EAT and thus does not represent a robust effect of chia oil supplementation or obesity. The only significant change occurred between depots in the HC group, with higher acetate secretion observed in EAT compared with SAT. This suggests that, under combined HFD and chia supplementation, visceral adipose tissue may handle acetate differently from subcutaneous fat, potentially due to intrinsic differences in carbon flux and acetyl‐CoA partitioning. However, because these changes were not observed across groups within each depot, acetate should be interpreted as a supportive but not central metabolic finding, complementary to the more consistent alterations seen in amino acid and lactate metabolism.

Omega‐3—rich oils can reshape adipose fuel selection by modulating the balance between glucose utilization, amino‐acid handling and acetyl‐CoA availability, ultimately influencing how carbon is partitioned between glycolysis, anaplerosis and lipid storage/turnover (Kalupahana et al. 2020; Gannon et al. 2018). In our explant model, the reduction in BCAA (valine) secretion and the coordinated shifts in amino acids linked to nitrogen/ECM‐related metabolism in SAT, together with increased lactate release in visceral EAT, support a depot‐specific reallocation of substrate handling under chia oil supplementation. Specifically, SAT appears to respond with greater flexibility in amino‐acid routing, whereas EAT shows a more prominent glycolytic output (lactate efflux), consistent with local metabolic programming rather than systemic lactate accumulation. The selective difference in acetate levels between EAT and SAT explants observed only in HC mice further suggests subtle depot‐dependent differences in acetyl‐CoA/acetate partitioning under combined HFD and chia oil exposure, even when net acetate release is not broadly altered across diets. Collectively, these patterns align with the idea that chia oil acts primarily by shifting adipose substrate portioning in a depot‐dependent manner, rather than producing uniform changes in single metabolite levels.

Consistent with this substrate reallocation, the correlation analysis revealed that chia oil supplementation induces network‐level metabolic remodeling, highlighting coordinated pathway shifts that are not captured by univariate metabolite changes alone. We emphasize that correlation analyses are associative and not mechanistic; however, they provide valuable insight into coordinated metabolic adjustments. In SAT, chia oil weakened canonical glycolytic associations—most notably the negative relationships between glucose–lactate and glucose–glutamate—suggesting a redirection of glucose carbon toward alternative metabolic routes, a phenomenon previously associated with omega‐3‐mediated modulation of glycolysis (Kalupahana et al. 2020). At the same time, chia oil strengthened correlations between BCAAs and between proline and BCAAs, indicating enhanced flexibility in amino‐acid utilization and potential modulation of pathways linked to insulin sensitivity and mTOR signaling (Gannon et al. 2018). The altered proline–glutamate correlation further suggests adaptations in ECM‐related amino‐acid handling, potentially reflecting reduced conversion of proline to glutamate.

In EAT, chia oil broadly disrupted glucose‐centered correlations present in both control and obese mice, including the strong negative glucose–glutamate association, which may reflect greater stabilization of substrate fluctuations and reduced metabolic stress in this depot. Chia supplementation also introduced several new positive correlations among BCAAs, proline, and glutamate, consistent with enhanced metabolic coordination and potentially supporting tissue remodeling or improved anabolic balance. Together, these depot‐specific shifts in correlation structure indicate that chia oil acts primarily by reorganizing metabolic interactions at the pathway level, rather than altering individual metabolites in isolation. This reconfiguration of metabolic crosstalk provides additional support for the notion that chia oil enhances the metabolic adaptability and functional plasticity of adipose tissue during obesity.

We acknowledge that ex vivo adipose explant culture may introduce metabolic stress and may not fully recapitulate in vivo dynamics. To account for baseline metabolites present in the culture medium, we analyzed the intact medium in parallel and interpreted conditioned‐media signals relative to this baseline. Although this approach controls for background and enables standardized comparisons across groups under identical incubation conditions, future studies incorporating time‐course sampling and/or complementary in vivo flux approaches will help refine the temporal dynamics of metabolite exchange and strengthen causal interpretation of pathway‐level changes.

This study used male mice only, which may limit the broader applicability of the findings. Future work should include females to evaluate potential sex‐specific responses to chia oil supplementation. We also recognize that sample size can influence metabolomics‐based inference. To mitigate false positives, univariate exometabolomic comparisons were interpreted under multiple‐testing control (FDR), and metabolite‐level findings were considered together with independent phenotypic outcomes (adipocyte morphometry and glucose tolerance) and targeted validation (lactate quantification and MCT expression). Overall, our conclusions are based on convergent evidence across complementary readouts rather than isolated metabolite differences.

While our study highlights important metabolic shifts induced by chia oil supplementation, its clinical implications remain to be fully established. The absence of significant changes in body weight suggests that its metabolic benefits may be related more to enhanced metabolic flexibility than to weight loss per se. Future studies should evaluate longer intervention windows and incorporate additional systemic readouts to better link depot‐specific exometabolic remodeling to whole‐body metabolic outcomes in obesity.

## 5 Conclusion

In conclusion, our pioneering study provides comprehensive insights into the intricate metabolic changes occurring within adipose tissue in response to obesity and dietary supplementation with chia oil. Using NMR‐based exometabolomics, we identified significant shifts in metabolite uptake and secretion, highlighting the interplay between glucose and amino acid metabolism in subcutaneous and epididymal adipose tissue. Chia oil supplementation appears to modulate the adipose tissue metabolic environment, influencing key metabolites involved in energy balance, amino acid metabolism, and inflammation, which may help mitigate obesity‐related complications. The altered metabolite correlations before and after supplementation highlight the dynamic nature of adipose tissue metabolism and the potential regulatory role of chia oil. These findings suggest potential targets for therapeutic exploration in the management of obesity and its associated comorbidities.

## Author Contributions

S.V.S. and G.C.S. designed research. A.A.‐F., G.M.‐C., T.E.B., and G.C.S. conducted research. S.V.S., C.B.‐F., and G.C.S. provided essential reagents and materials. S.V.S., A.A.‐F., and G.C.S. analyzed data and performed statistical analysis. S.V.S. and G.C.S. wrote paper. S.V.S. had primary responsibility for final content. All authors have read and approved the final manuscript.

## Funding

This work was supported by the Conselho Nacional de Desenvolvimento Científico e Tecnológico (Project No. 444675/2023‐0; 402918/2023‐1), Fundação Carlos Chagas Filho de Amparo à Pesquisa do Estado do Rio de Janeiro (Project No. SEI‐260003/001184/2023; SEI‐260003/005820/2024; E26/201.259/2021/JCNE). The funders had no role in the study design, data collection, data analysis, preparation of the manuscript, or the decision to publish.

## Conflicts of Interest

The authors declare no conflicts of interest.

## Supporting information


**Figure S1:** Representative NMR spectra showing metabolite peaks identified in conditioned media from SAT and EAT explants.
**Figure S2:** Volcano plots illustrate statistically significant metabolite alterations induced by chia oil supplementation.
**Figure S3:** Lactate levels and expression of monocarboxylate transporters (MCT1 and MCT4) in serum, subcutaneous adipose tissue (SAT), and epididymal adipose tissue (EAT). (A) Serum lactate concentrations showed no significant differences among groups. (B) In SAT, lactate levels were significantly lower in CC and H compared with C. (C, D) Gene expression of MCT1 and MCT4 in SAT. MCT1 expression was unchanged across groups, whereas MCT4 was significantly upregulated in CC relative to C, H, and HC. (E) In EAT, lactate release was significantly higher in HC compared with H, indicating depot‐specific modulation of glycolytic output. (F, G) Gene expression of MCT1 and MCT4 in EAT showed no significant differences among groups, suggesting that changes in lactate release from EAT are not driven by transcriptional regulation of these transporters. Data are presented as mean ± SEM.
**Figure S4:** Spearman correlation heatmap detailing correlations and respective *p*‐values for metabolites analyzed in SAT.
**Figure S5:** Spearman correlation heatmap detailing correlations and respective *p*‐values for metabolites analyzed in EAT.
**Table S1:** Diet composition.
**Table S2:** Fatty acid composition of diets.
**Table S3:** Genes analyzed by qPCR.

**Table S4:** (XLSX): List of metabolites identified by NMR‐based metabolomics, along with their chemical shifts (ppm).

## Data Availability

The data that support the findings of this study are openly available in Metabolomics Workbench at https://www.metabolomicsworkbench.org/data/databases.php.
