# Supplementary material for: Exometabolomics Reveals Metabolic Modulation Induced by Chia Oil Supplementation in Adipose Tissue Explants From Obese Mice
Source: Lipids. 2026 May 31;61(6):675–89. doi: 10.1002/lipd.70066 (PMC13751177; doi:10.1002/lipd.70066)
Supplement: Supplementary file 1 [file LIPD-61-675-s001.docx]

**Exometabolomics reveals metabolic modulation induced by chia oil supplementation in adipose tissue explants from obese mice**

Gilson Costa dos Santos Jr^1^; Agatha Assis-Ferreira^2^; Gabrielly Muniz-Cassuce^2^; Tatiana El Bacha^3^; Christina Barja-Fidalgo^2^; Simone Vargas da Silva^2*^

1. Laboratório de Metabolômica; Departamento de Genética, Instituto de Biologia Roberto Alcântara Gomes, Universidade do Estado do Rio de Janeiro, RJ, Brazil.
2. Laboratório de Farmacologia Celular e Molecular, Departamento de Biologia Celular, Instituto de Biologia Roberto Alcântara Gomes, Universidade do Estado do Rio de Janeiro, RJ, Brazil.
3. Núcleo de Estudos com Bioativos, Mitocôndria e Metabolismo da Placenta, Instituto de Nutrição Josué de Castro, Universidade Federal do Rio de Janeiro, RJ, Brazil.

* Correspondence: [simonevargasdasilva@gmail.com](mailto:simonevargasdasilva@gmail.com)

# Table of Contents

**Supplementary Figure S1**: Representative NMR spectra showing metabolite peaks identified in conditioned media from SAT and EAT explants.

**Supplementary Figure S2**: Volcano plots illustrating statistically significant metabolite alterations induced by chia oil supplementation.

**Supplementary Figure S3**: Lactate levels and expressions of monocarboxylate transporters (MCT1 and MCT4) in serum, subcutaneous adipose tissue (SAT), and epididymal adipose tissue (EAT).

**Supplementary Figure S4 and S5**: Spearman correlation heatmap detailing correlations and respective p-values for metabolites analyzed in SAT and EAT.

**Supplementary Table S1**: Diet composition

**Supplementary Table S2**: Fatty acid composition of diets

**Supplementary Table S3**: Genes analyzed by qPCR.

**Supplementary Table 4 (XLSX)**: List of metabolites identified by NMR-based metabolomics, along with their chemical shifts (ppm).

## Supplementary Figure S1

Supplementary Figure S1. Representative NMR spectra showing metabolite peaks identified in conditioned media from SAT and EAT explants.

## Supplementary Figure S2

Supplementary Figure S2. Volcano plots illustrate statistically significant metabolite alterations induced by chia oil supplementation.

## Supplementary Figure S3

Supplementary Figure S3: Lactate levels and expression of monocarboxylate transporters (MCT1 and MCT4) in serum, subcutaneous adipose tissue (SAT), and epididymal adipose tissue (EAT). (A) Serum lactate concentrations showed no significant differences among groups. (B) In SAT, lactate levels were significantly lower in CC and H compared with C. (C–D) Gene expression of MCT1 and MCT4 in SAT. MCT1 expression was unchanged across groups, whereas MCT4 was significantly upregulated in CC relative to C, H, and HC. (E) In EAT, lactate release was significantly higher in HC compared with H, indicating depot-specific modulation of glycolytic output. (F–G) Gene expression of MCT1 and MCT4 in EAT showed no significant differences among groups, suggesting that changes in lactate release from EAT are not driven by transcriptional regulation of these transporters. Data are presented as mean ± SEM.

## Supplementary Figure S4

**Subcutaneous adipose Tissue**

**C**

|  | Lactate | Glucose | Glutamate | Leucine | Valine | Threonine | Proline | Histidine |
| --- | --- | --- | --- | --- | --- | --- | --- | --- |
| Lactate |  | **0.0167** | 0.0833 | 0.4333 | 0.9500 | 0.6333 | 0.9500 | 0.6833 |
| Glucose | **0.0167** |  | 0.0833 | 0.4333 | 0.9500 | 0.6333 | 0.9500 | 0.6833 |
| Glutamate | 0.0833 | 0.0833 |  | 0.6667 | 0.6833 | 0.9000 | 0.6833 | 0.9500 |
| Leucine | 0.4333 | 0.4333 | 0.6667 |  | 0.4333 | 0.2000 | 0.4333 | 0.8333 |
| Valine | 0.9500 | 0.9500 | 0.6833 | 0.4333 |  | 0.1333 | 0.1333 | 1.0000 |
| Threonine | 0.6333 | 0.6333 | 0.9000 | 0.2000 | 0.1333 |  | 0.0667 | 0.6333 |
| Proline | 0.9500 | 0.9500 | 0.6833 | 0.4333 | 0.1333 | 0.0667 |  | 0.3500 |
| Histidine | 0.6833 | 0.6833 | 0.9500 | 0.8333 | 1.0000 | 0.6333 | 0.3500 |  |

**CC**

|  | Lactate | Glucose | Glutamate | Leucine | Valine | Threonine | Proline | Histidine |
| --- | --- | --- | --- | --- | --- | --- | --- | --- |
| Lactate |  | **0.0277** | 0.0761 | 0.0609 | 0.4548 | 0.2687 | 0.4720 | 0.5702 |
| Glucose | **0.0277** |  | **0.0004** | 0.0708 | 0.2884 | 0.3511 | **0.0339** | 0.4054 |
| Glutamate | 0.0761 | **0.0004** |  | 0.4023 | 0.7905 | 0.4800 | **0.0099** | 0.5069 |
| Leucine | 0.0609 | 0.0708 | 0.4023 |  | **0.0137** | 0.1426 | 0.6873 | 0.6083 |
| Valine | 0.4548 | 0.2884 | 0.7905 | **0.0137** |  | 0.1939 | 0.4027 | 0.4105 |
| Threonine | 0.2687 | 0.3511 | 0.4800 | 0.1426 | 0.1939 |  | 0.5971 | 0.6855 |
| Proline | 0.4720 | **0.0339** | **0.0099** | 0.6873 | 0.4027 | 0.5971 |  | 0.3869 |
| Histidine | 0.5702 | 0.4054 | 0.5069 | 0.6083 | 0.4105 | 0.6855 | 0.3869 |  |

**H**

|  | Lactate | Glucose | Glutamate | Leucine | Valine | Threonine | Proline | Histidine |
| --- | --- | --- | --- | --- | --- | --- | --- | --- |
| Lactate |  | 0.4697 | 0.9528 | 0.5289 | 0.4271 | 0.9381 | 0.1139 | 0.2732 |
| Glucose | 0.4697 |  | **0.0497** | 0.3910 | 0.2325 | 0.8570 | 0.0544 | 0.8989 |
| Glutamate | 0.9528 | **0.0497** |  | 0.5975 | **0.0065** | 0.1097 | 0.2988 | 0.5538 |
| Leucine | 0.5289 | 0.3910 | 0.5975 |  | 0.2273 | **0.0278** | 0.9803 | 0.8820 |
| Valine | 0.4271 | 0.2325 | **0.0065** | 0.2273 |  | **0.0206** | 0.5367 | 0.5890 |
| Threonine | 0.9381 | 0.8570 | 0.1097 | **0.0278** | **0.0206** |  | 0.9923 | 0.3616 |
| Proline | 0.1139 | 0.0544 | 0.2988 | 0.9803 | 0.5367 | 0.9923 |  | 0.0743 |
| Histidine | 0.2732 | 0.8989 | 0.5538 | 0.8820 | 0.5890 | 0.3616 | 0.0743 |  |

**HC**

|  | Lactate | Glucose | Glutamate | Leucine | Valine | Threonine | Proline | Histidine |
| --- | --- | --- | --- | --- | --- | --- | --- | --- |
| Lactate |  | 0.8987 | 0.9910 | 0.4269 | 0.0989 | 0.4226 | **0.0428** | 0.1627 |
| Glucose | 0.8987 |  | 0.3813 | **0.0075** | 0.3997 | 0.1123 | 0.4664 | 0.3731 |
| Glutamate | 0.9910 | 0.3813 |  | 0.9916 | 0.0556 | 0.5060 | 0.0750 | 0.2242 |
| Leucine | 0.4269 | **0.0075** | 0.9916 |  | 0.0816 | **0.0096** | **0.0409** | 0.7856 |
| Valine | 0.0989 | 0.3997 | 0.0556 | 0.0816 |  | 0.0869 | **0.0001** | 0.9083 |
| Threonine | 0.4226 | 0.1123 | 0.5060 | **0.0096** | 0.0869 |  | **0.0085** | 0.7358 |
| Proline | **0.0428** | 0.4664 | 0.0750 | **0.0409** | **0.0001** | **0.0085** |  | 0.6231 |
| Histidine | 0.1627 | 0.3731 | 0.2242 | 0.7856 | 0.9083 | 0.7358 | 0.6231 |  |

Supplementary Figure S4. Spearman correlation heatmap detailing correlations and respective p-values for metabolites analyzed in SAT.

**Supplementary Figure S5**

**C**

|  | Lactate | Glucose | Glutamate | Leucine | Valine | Threonine | Proline | Histidine |
| --- | --- | --- | --- | --- | --- | --- | --- | --- |
| Lactate |  | 1.0000 | 0.6615 | 0.1698 | 0.3956 | 0.3159 | 1.0000 | 0.6615 |
| Glucose | 1.0000 |  | **0.0238** | 0.7976 | 0.3956 | 0.4008 | **0.0413** | **0.0480** |
| Glutamate | 0.6615 | **0.0238** |  | 0.4817 | 0.1389 | 0.6230 | **0.0040** | 0.2667 |
| Leucine | 0.1698 | 0.7976 | 0.4817 |  | 0.2048 | 0.6972 | 0.5222 | 0.3183 |
| Valine | 0.3956 | 0.3956 | 0.1389 | 0.2048 |  | 0.9508 | 0.2548 | 0.7131 |
| Threonine | 0.3159 | 0.4008 | 0.6230 | 0.6972 | 0.9508 |  | 0.8433 | 0.5373 |
| Proline | 1.0000 | **0.0413** | **0.0040** | 0.5222 | 0.2548 | 0.8433 |  | 0.2183 |
| Histidine | 0.6615 | **0.0480** | 0.2667 | 0.3183 | 0.7131 | 0.5373 | 0.2183 |  |

**CC**

|  | Lactate | Glucose | Glutamate | Leucine | Valine | Threonine | Proline | Histidine |
| --- | --- | --- | --- | --- | --- | --- | --- | --- |
| Lactate |  | 0.8846 | 0.9950 | 0.9929 | 0.4799 | 0.2210 | 0.7807 | 0.8019 |
| Glucose | 0.8846 |  | 0.3896 | **0.0222** | 0.5804 | 0.8666 | 0.8870 | 0.3896 |
| Glutamate | 0.9950 | 0.3896 |  | 0.6783 | **0.0226** | 0.3082 | **0.0440** | 0.6538 |
| Leucine | 0.9929 | **0.0222** | 0.6783 |  | **0.0228** | 0.6756 | 0.2814 | 0.5997 |
| Valine | 0.4799 | 0.5804 | **0.0226** | **0.0228** |  | 0.2441 | **0.0226** | 0.6337 |
| Threonine | 0.2210 | 0.8666 | 0.3082 | 0.6756 | 0.2441 |  | 0.4720 | 0.1944 |
| Proline | 0.7807 | 0.8870 | **0.0440** | 0.2814 | **0.0226** | 0.4720 |  | 0.3415 |
| Histidine | 0.8019 | 0.3896 | 0.6538 | 0.5997 | 0.6337 | 0.1944 | 0.3415 |  |

**H**

|  | Lactate | Glucose | Glutamate | Leucine | Valine | Threonine | Proline | Histidine |
| --- | --- | --- | --- | --- | --- | --- | --- | --- |
| Lactate |  | 0.6257 | 0.2349 | 0.6341 | 0.0520 | 0.0689 | 0.9772 | 0.9923 |
| Glucose | 0.6257 |  | 0.5467 | 0.8733 | 0.9662 | 0.8396 | 0.1882 | 0.3308 |
| Glutamate | 0.2349 | 0.5467 |  | 0.8549 | **0.0096** | 0.7618 | 0.1430 | 0.5652 |
| Leucine | 0.6341 | 0.8733 | 0.8549 |  | 0.2207 | 0.1395 | 0.5614 | 0.7393 |
| Valine | 0.0520 | 0.9662 | **0.0096** | 0.2207 |  | 0.1864 | 0.3911 | 0.8382 |
| Threonine | 0.0689 | 0.8396 | 0.7618 | 0.1395 | 0.1864 |  | 0.8014 | 0.4202 |
| Proline | 0.9772 | 0.1882 | 0.1430 | 0.5614 | 0.3911 | 0.8014 |  | 0.3067 |
| Histidine | 0.9923 | 0.3308 | 0.5652 | 0.7393 | 0.8382 | 0.4202 | 0.3067 |  |

**HC**

|  | Lactate | Glucose | Glutamate | Leucine | Valine | Threonine | Proline | Histidine |
| --- | --- | --- | --- | --- | --- | --- | --- | --- |
| Lactate |  | 0.8861 | 0.4386 | 0.6550 | 0.4708 | 0.6389 | 0.9433 | 0.6999 |
| Glucose | 0.8861 |  | **0.0310** | 0.0996 | 0.5731 | 0.4465 | 0.2240 | 0.3194 |
| Glutamate | 0.4386 | **0.0310** |  | 0.7460 | 0.1215 | 0.5279 | **0.0017** | 0.5833 |
| Leucine | 0.6550 | 0.0996 | 0.7460 |  | 0.2111 | **0.0055** | 0.6391 | 0.1050 |
| Valine | 0.4708 | 0.5731 | 0.1215 | 0.2111 |  | 0.1816 | **0.0351** | 0.4434 |
| Threonine | 0.6389 | 0.4465 | 0.5279 | **0.0055** | 0.1816 |  | 0.4579 | 0.3156 |
| Proline | 0.9433 | 0.2240 | **0.0017** | 0.6391 | **0.0351** | 0.4579 |  | **0.0362** |
| Histidine | 0.6999 | 0.3194 | 0.5833 | 0.1050 | 0.4434 | 0.3156 | **0.0362** |  |

Supplementary Figure S5. Spearman correlation heatmap detailing correlations and respective p-values for metabolites analyzed in EAT.

## Supplementary Table S1: Diet composition

| **Ingredient** | **C** | **CC** | **H** | **HC** |
| --- | --- | --- | --- | --- |
| **Corn starch** | 39 | 39 | 18.5 | 18.5 |
| **Dextrose** | 13.2 | 13.2 | 6.1 | 6.1 |
| **Casein** | 20 | 20 | 20 | 20 |
| **Sucrose** | 10 | 10 | 10 | 10 |
| **Soybean oil** | 7 | 7 | 7 | 7 |
| **Cellulose** | 5 | 5 | 5 | 5 |
| **Mineral mix** | 3.5 | 3.5 | 3.5 | 3.5 |
| **Vitamin mix** | 1 | 1 | 1 | 1 |
| **Cystine** | 0.3 | 0.3 | 0.3 | 0.3 |
| **Choline** | 0.25 | 0.25 | 0.25 | 0.25 |
| **Lard** | 0 | 0 | 13.3 | 13.3 |
| **Chia oil** | 0 | 1.5 | 0 | 1.5 |
| **Carbohydrates** | 63 | 63 | 34 | 34 |
| **Proteins** | 20 | 20 | 20 | 20 |
| **Lipids** | 7 | 8.5 | 20.2 | 21.7 |
| **Energy (kcal/g)** | 3.96 | 4.09 | 4.73 | 4.81 |

Notes: C, control diet; CC, control diet supplemented with chia oil; H, high-fat diet; HC, high-fat diet supplemented with chia oil. Chia oil was incorporated into the diet at 1.5g/100g of diet (1.5%, w/w). Values are expressed as grams per 100 g of diet (g/100 g). Energy density (kcal/g) was calculated based on standard factors (4 kcal/g for carbohydrates and proteins; 9 kcal/g for lipids).

**Supplementary Table S2. Fatty acid composition of the diets**

| **Fatty acid (g/100 g)** | **C** | **CC** | **H** | **HC** |
| --- | --- | --- | --- | --- |
| **Palmitic (16:0)** | 0.75 | 0.84 | 3.46 | 3.55 |
| **Stearic (18:0)** | 0.23 | 0.28 | 1.67 | 1.72 |
| **Oleic (18:1 ω-9)** | 1.60 | 1.69 | 6.76 | 6.85 |
| **Linoleic (18:2 ω-6)** | 3.76 | 4.05 | 6.34 | 6.15 |
| **α-linolenic (18:3 ω-3)** | 0.40 | 1.37 | 0.55 | 1.5 |
| **SFA** | 1.06 | 1.20 | 6.31 | 6.45 |
| **PUFA** | 4.20 | 5.42 | 12.14 | 13.36 |
| **ω-6:ω-3 ratio** | 9.40 | 2.95 | 11.52 | 4.1 |

Notes: C, control diet; CC, control diet supplemented with chia oil; H, high-fat diet; HC, high-fat diet supplemented with chia oil. SFA – saturated fatty acids, PUFA – polyunsaturated fatty acids. Incorporation of 1.5% chia oil in the CC and HC diets increased α- linolenic acid content and reduced the ω-6:ω-3 ratio compared to C and H diet, respectively, resulting in a more balanced fatty acid profile consistent with nutritional recommendations favoring lower ω-6:ω-3 ratios.

## Supplementary Table S3: Genes analyzed by qPCR

| Gene Symbol | Function/Description | Access number of Gene bank | Catalog number |
| --- | --- | --- | --- |
| rn18S | Housekeeping gene for normalization | NR003278 | QT02448075 |
| Slc16a1 (MCT1) | Monocarboxylic acid transporters, member 1 | NM009196 | QT00115423 |
| Slc16a4 (MCT4) | Monocarboxylic acid transporters, member 4 | NM146136 | QT00116039 |

## Notes: Primers used for gene expression analysis by quantitative real-time PCR. Primers were purchased as QuantiTect Primer Assays from Qiagen® (Germany), optimized for SYBR Green-based detection. The rn18S gene was used as the endogenous control for normalization.

## Supporting Table S4 (Provided separately as XLSX)

List of metabolites identified by NMR-based metabolomics, along with their chemical shifts (ppm).
